# Whole-genome enrichment and sequencing of *Chlamydia trachomatis*directly from clinical samples

**DOI:** 10.1186/s12879-014-0591-3

**Published:** 2014-11-12

**Authors:** Mette T Christiansen, Amanda C Brown, Samit Kundu, Helena J Tutill, Rachel Williams, Julianne R Brown, Jolyon Holdstock, Martin J Holland, Simon Stevenson, Jayshree Dave, CY William Tong, Katja Einer-Jensen, Daniel P Depledge, Judith Breuer

**Affiliations:** Division of Infection and Immunity University College London (UCL), London, WC1E 6BT UK; Oxford Gene Technology, Begbroke, OX5 1PF Oxfordshire, UK; Great Ormond Street Hospital (GOSH), London, WC1N 3JH UK; London School of Hygiene and Tropical Medicine (LSHTM), London, WC1E 7HT UK; University College London Hospital (UCLH), London, WC1E 6DE UK; Barts Health NHS Trust, London, E1 2ES UK; QIAGEN-AAR, Aarhus N, 8200 Denmark; Department of Microbiology and Immunology, Cornell University, Ithaca, 14853 NY USA; School of Human and Life Sciences, Canterbury Christchurch University, CT1 1QU Canterbury, Kent, UK

**Keywords:** Whole-genome enrichment, Whole-genome sequencing, Chlamydia trachomatis, Clinical samples

## Abstract

**Background:**

*Chlamydia trachomatis* is a pathogen of worldwide importance, causing more than 100 million cases of sexually transmitted infections annually. Whole-genome sequencing is a powerful high resolution tool that can be used to generate accurate data on bacterial population structure, phylogeography and mutations associated with antimicrobial resistance. The objective of this study was to perform whole-genome enrichment and sequencing of *C. trachomatis* directly from clinical samples.

**Methods:**

*C. trachomatis* positive samples comprising seven vaginal swabs and three urine samples were sequenced without prior *in vitro* culture in addition to nine cultured *C. trachomatis* samples, representing different serovars. A custom capture RNA bait set, that captures all known diversity amongst *C. trachomatis* genomes, was used in a whole-genome enrichment step during library preparation to enrich for *C. trachomatis* DNA. All samples were sequenced on the MiSeq platform.

**Results:**

Full length *C. trachomatis* genomes (>95-100% coverage of a reference genome) were successfully generated for eight of ten clinical samples and for all cultured samples. The proportion of reads mapping to *C. trachomatis* and the mean read depth across each genome were strongly linked to the number of bacterial copies within the original sample. Phylogenetic analysis confirmed the known population structure and the data showed potential for identification of minority variants and mutations associated with antimicrobial resistance. The sensitivity of the method was >10-fold higher than other reported methodologies.

**Conclusions:**

The combination of whole-genome enrichment and deep sequencing has proven to be a non-mutagenic approach, capturing all known variation found within *C. trachomatis* genomes. The method is a consistent and sensitive tool that enables rapid whole-genome sequencing of *C. trachomatis* directly from clinical samples and has the potential to be adapted to other pathogens with a similar clonal nature.

**Electronic supplementary material:**

The online version of this article (doi:10.1186/s12879-014-0591-3) contains supplementary material, which is available to authorized users.

## Background

*Chlamydia trachomatis* is the most common bacterial agent in sexually transmitted infections (STI), globally accounting for more than 100 million infections [1],[2]. This bacterium (serovar A-C) causes the blinding disease, trachoma, which affects millions of people worldwide [3],[4]. The morbidity from *C. trachomatis* infections places a heavy economic burden on society [5],[6].

*C. trachomatis* is an obligate intracellular pathogen with a two-phase developmental cycle that consists of infectious elementary bodies (EBs) and replicating reticulate bodies (RBs). EBs are taken up by host cells into an inclusion vacuole in which they differentiate into replicating RBs. The cycle ends when the RBs differentiate back into EBs and are released from the host cell by lysis or egress of inclusions [7],[8]. EBs have traditionally been described as metabolic inactive but a recent study has shown metabolic activity in EBs when cultured in a laboratory cell-free system [9].

*C. trachomatis* strains are classified into two biovars: the ocular/urogenital biovar and the lymphogranuloma venereum (LGV) biovar (for review) [10]. The two biovars can be subdivided into 15-19 different serovars. Further genotypic classification is based on nucleotide sequencing of the *ompA* gene, which encodes the major outer membrane protein and is the target of serotype classification. The ocular/urogenital biovar consists of the ocular serovars A-C and the urogenital serovars D-K, all of which are usually confined to mucosal epithelia whereas the LGV biovar, consisting of serotypes L1-L3, is more invasive and can disseminate to other tissues and the draining lymphatic system. It has previously been demonstrated that genotyping of the *ompA* gene is insufficient for exploring *C. trachomatis* population structure and performing molecular epidemiological studies on transmission as this region undergoes high levels of recombination [11]. Also, variation within the *ompA* gene differs among serovars and sexual networks can be predominated by a single serovar, making strain distribution and evolutionary studies impractical [12],[13]. In this context, whole genome sequencing (WGS) has been used to generate accurate data on bacterial population structure and phylogeography [14]. In addition whole genome sequencing can also facilitate the identification of mutations associated with antimicrobial resistance [15],[16].

Clinical samples often contain low numbers of pathogens and to obtain sufficient material for WGS of *C. trachomatis*, *in vitro* culture is usually required. However, as *C. trachomatis* is an obligate intracellular pathogen it is labour intensive to grow *in vitro*[17]. For this reason, methods that allow sequencing directly from *C. trachomatis* positive samples are particularly attractive. An example of this was recently described [18]; the approach was based on an antibody-based enrichment step targeting intact *C. trachomatis* cells followed by whole genome amplification of the total amount of DNA within the sample. The method proved useful for sequencing *C. trachomatis* from complex clinical samples but required in excess of 1,500,000 genome copies per microliter, following whole genome amplification, and sequencing on an Illumina HiSeq to generate sufficient numbers of reads for *C. trachomatis* genome mapping. Overall this approach showed only a 15-30% success rate, which underlines the need for a more reliable methodology.

It has previously been shown that the SureSelect^XT^ Target-Enrichment protocol (Agilent Technologies), which uses custom designed 120-mer RNA oligonucleotides that span the entire genome, can recover (by hybridisation) low copy number herpesviruses from clinical samples with sufficiently high sensitivity and specificity to enable ultra-deep whole genome sequencing [19],[20]. In this study we used the SureSelect^XT^ Target-Enrichment approach to improve the sensitivity of *C. trachomatis* whole genome sequencing from clinical specimens. This method offers the opportunity for gaining a wider understanding of the *C. trachomatis* population structures, transmission patterns and of the evolution of antimicrobial resistance.

## Methods

### Ethics statement

The clinical samples were obtained independently from patients with confirmed genital *Chlamydia trachomatis* infections. These were diagnostics samples collected as part of the standard clinical procedure at Barts Health NHS Trust and were obtained by the UCL Infection DNA Bank for use in this study. All samples were supplied to the study in an anonymised form and the use of these specimens for research was approved by the NRES Committee London - Fulham (REC reference: 12/LO/1089).

### *C. trachomatis*culture samples

Nine isolates were obtained from a sample archive at University College London Hospital (UCLH). These cultured isolates were used to optimise the bait set. Originally these *C. trachomatis* samples were obtained from clinical specimens and isolated via several embryonated chick egg passages as described previously [21]. The samples were stored at −80°C after original isolation. After several years of storage the samples were propagated in cycloheximide treated McCoy cells (without antibiotics) in shell vials and then inoculated on to 25 cm^2^ tissue culture flasks. After the second round of culture the samples were harvested, centrifuged and re-suspended in Minimum Essential Medium (MEM) supplemented with Hank's balanced salt solution, L glutamine and dimethyl sulfoxide (DMSO) as a cryo-preservative and stored at −80°C until DNA extraction.

For DNA extraction, the samples were thawed and the cells were pelleted, re-suspended in 100 μl proteinase K solution and incubated at 60°C for 1 hour. DNA was extracted from the samples using the Promega Wizard Genomic DNA Purification Kit. *C. trachomatis* DNA within each sample was quantified by qPCR targeting the *C. trachomatis* plasmid and the genomic *omcB* gene, using a dilution series of plasmid containing the target sequence as standard. Human RNase-P was used as an endogenous control.

### *C. trachomatis c*linical samples

Ten *C. trachomatis* diagnostic positive samples (tested using the Viper platform, BD Diagnostics) were collected at Barts Health NHS Trust: seven vaginal swabs and three urine samples. The samples were stored at −80°C until DNA extraction. DNA was extracted directly from clinical specimens, without culture, using the QIAGEN QIAamp DNA Mini Kit. *C. trachomatis* DNA within each sample was quantified by qPCR, targeting the *C. trachomatis* plasmid and the genomic *omcB* gene. Human RNase-P was used as an endogenous control [22].

### SureSelect^XT^Target Enrichment: RNA baits design

The 120-mer RNA baits spanning the length of the positive strand of 74 GenBank *C. trachomatis* reference genomes were designed using an in-house PERL script developed by the PATHSEEK consortium. The specificity of the baits was verified by BLASTn searches against the Human Genomic + Transcript database. The custom designed *C. trachomatis* bait library was uploaded to SureDesign and synthesised by Agilent Technologies.

### SureSelect^**XT**^Target Enrichment: Library preparation, hybridisation and enrichment

*C. trachomatis* DNA samples were quantified and carrier human genomic DNA (Promega) was added to obtain a total of 3 μg input for library preparation. To assess the sensitivity of the whole-genome enrichment and sequencing a dilution series of *C. trachomatis* DNA extracted from culture was generated. One undiluted sample and 6 dilutions were generated with target input DNA (*C. trachomatis* DNA) ranging from 3 μg to 0.01 ng. The diluted samples were bulked to 3 μg with human carrier DNA, as before.

All DNA samples were sheared for 6x60 seconds using a Covaris E210 (duty cycle 10%, intensity 5 and 200 cycles per burst using frequency sweeping). End-repair, non-templated addition of 3’ poly A, adapter ligation, hybridisation, PCR and all post- reaction clean-up steps were performed according to the SureSelect^XT^ Illumina Paired-End Sequencing Library protocol (V1.4.1 Sept 2012). All recommended quality control steps were performed.

### Illumina sequencing

Samples were multiplexed to combine either eight or ten samples per run. Paired end sequencing was performed on an Illumina MiSeq sequencing platform with a 300 bp v2 reagent set. Base calling and sample demultiplexing were generated as standard producing paired FASTQ files for each sample. The cultured samples were sequenced in a single run whereas the sequencing of the clinical samples was repeated to increase sequence depth.

### Sequence data analysis

Genome mapping, assembly and finishing was performed using CLC Genomics Workbench (version 6.5.0/6.5.1) including the CLC Microbial Genome Finishing Module (version 1.2.1/1.3.0) from Qiagen. For each data set, all read-pairs were subject to quality control and reads were quality trimmed based on the presence of ambiguous nucleotides using the default parameters (Additional file 1). All remaining reads were mapped to a *C. trachomatis* reference genome and consensus sequences extracted using default parameters. Samples from the ocular/urogenital biovar (ten clinical samples plus seven cultured samples) were aligned to *C. trachomatis* isolate F/SW4 (GenBank accession no. NC_017951.1) whereas samples from the LGV biovar (two cultured samples) were aligned to *C. trachomatis* L2/434/Bu reference genome (GenBank accession no. AM884176). The urogenital strain F/SW4 has been used as reference in a previous study where it was defined as a completed high-quality reference [18] and was applied here due to the urogenital nature, which is compatible with the clinical sample set obtained from vaginal swabs (female patients) and urine (male patients). When defining mean read depth obtained from the *C. trachomatis* from vaginal swabs, the duplicated rRNA regions were masked as these regions were found to have a significantly higher read depth compared to the rest of the *C. trachomatis* genome (see coverage plot in Additional file 1). For comparison, *de novo* assembly was also performed for the clinical samples. All assemblies were performed using CLC Genomics Workbench with default parameters. The Microbial Genome Finishing Module was applied for *de novo* assembly in CLC Genomics Workbench and mapping mode was set to `map reads back to contigs (slow). Following reference based assembly, *in silico* genotyping was performed on the *ompA* gene sequences obtained from the clinical samples. Consensus sequences were aligned with *C. trachomatis* whole genome sequences found in GenBank using MAFFT (progressive approach) and the alignment was visualised and manually corrected in MEGA [23],[24]. SNP differences between a cultured sample, which was processed with and without whole-genome enrichment, and the *C. trachomatis* isolate F/SW4 (GenBank accession no. NC_017951.1) were called with Base-By-Base and SNPs found within coding regions were visualised in a Circos-plot [25],[26]. To assess any potential differences associated with body compartments, non-synonymous SNP difference between the clinical consensus sequences and the reference (*C. trachomatis* isolate F/SW4) were identified and visualized in a Circos plot [26].

Mutations in the *C. trachomatis* genome associated with antibiotic resistance were identified from the literature [16],[27]-[33]. The mapping data from the clinical samples were assessed for these mutations.

For samples with high mean read depth, variants were called using quality-based variant detection in CLC Genomics Workbench with a minimum read depth of 40x, minimum average quality of 20, and minimum required variant count of 2 present on both the forward and the reverse reads with a strand bias interval between 20-80% [20],[34]. Variant sites with frequencies >5% were inspected manually.

Phylogenetic reconstructions of the alignment data were performed and neighbour-joining trees were generated using various models of evolution and a gamma correction for among-site variation with four rate categories using MEGA [24]. The appropriate model of substitution for generation of a maximum likelihood tree was identified using jModelTest and a maximum likelihood tree was built using RAxML [35],[36]. All trees were generated with 500 bootstrap replicates.

Recombination within the *ompA* gene was evaluated for all the clinical samples using RAT, SBP and GARD [37],[38].

The sequence data from the clinical samples was submitted to the National Center for Biotechnology Information (NCBI) Sequence Read Archive (SRA) database as Bioproject accession PRJNA262506.

## Results

We sequenced 19 *C. trachomatis* samples, 9 from culture material and 10 directly from clinical material; three urine samples and seven vaginal swabs.

**Figure 1:**
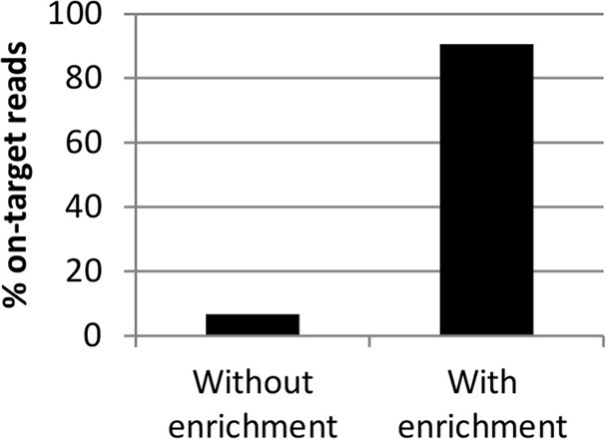
**The effect of whole-genome enrichment on the proportion of reads mapping to** ***C. trachomatis.*** The plot shows the proportion of reads mapping to a *C. trachomatis* reference (F/SW4) genome (% on-target reads) from a *C. trachomatis* cultured sample that was processed with and without whole-genome enrichment.

To assess whether whole-genome enrichment increases the number of reads mapping to the *C. trachomatis* genome, extracted DNA from a single *C. trachomatis* cultured sample was processed with and without whole-genome enrichment prior to sequencing. All sequence reads generated from the two libraries were mapped to a reference genome (isolate F/SW4 GenBank accession NC_017951.1) and the proportions of sequence reads mapping calculated. With whole-genome enrichment ~91% of the sequence reads mapped to *C. trachomatis* whereas without enrichment only ~7% of the sequence reads mapped to *C. trachomatis* (Figure 1).

**Figure 2:**
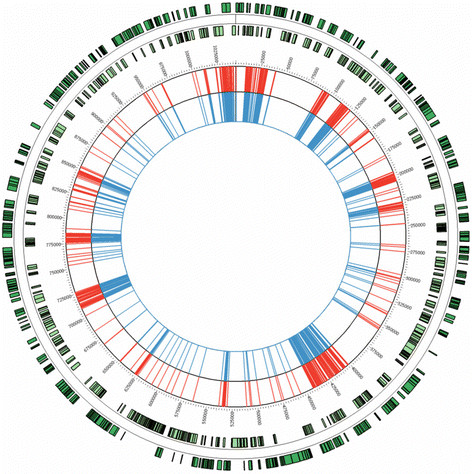
**SNP profiles in a** ***C. trachomatis*** **sample processed with and without whole-genome enrichment.** The plot illustrates SNP profiles in a cultured *C. trachomatis* sample processed with and without whole-genome enrichment compared to the GenBank reference strain isolate F/SW4 (Accession no. NC_017951.1). The two outer tracks shown in green illustrate the open reading frames (ORFs) annotated in the GenBank reference strain isolate F/SW4 with forward and reverse orientation. The red track shows the SNP differences found within coding regions between the cultured *C. trachomatis* sample processed without whole-genome enrichment and the reference strain. The blue track shows the SNP differences found within coding regions between the same cultured *C. trachomatis* sample processed with whole-genome enrichment and the reference strain. The SNPs are called at the consensus level.

To evaluate if whole-genome enrichment introduces any mutational bias, we compared the single nucleotide polymorphic differences (SNPs - single nucleotide polymorphisms) found between the consensus sequence recovered from the sample processed with enrichment and the consensus sequence recovered from the same sample processed without and the reference strain (F/SW4). Overall, no differences in the SNP profiles were found at any position in the genome (both coding and non-coding position) indicating that no mutational bias is introduced by the whole-genome enrichment method used (Figure 2 – for visualization only SNP differences within coding regions are included in the circos plot).

**Figure 3:**
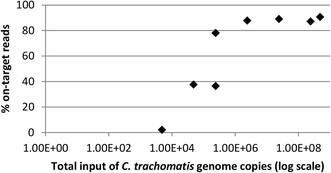
**Proportion of reads mapping to** ***C. trachomatis*** **reference genome in relation to total** ***C. trachomatis*** **genome copies input.** Plot showing the relationship between the numbers of input *C. trachomatis* genomes calculated and the proportion of reads mapping to the *C. trachomatis* reference (F/SW4) genome (% on-target reads).

**Figure 4:**
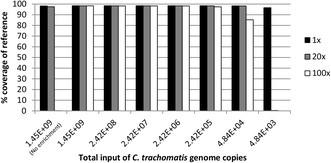
**Coverage of reference genome and mean read depth in relation to total** ***C. trachomatis*** **genome copies input.** The plot shows the amount of target DNA (*C. trachomatis* DNA) used in the library preparations and the percentage coverage of the reference genome obtained with the various target input. The bars (black, grey, and white) illustrate the minimum mean read depth obtained from each sample. The columns to the far left represent a sample which did not undergo whole-genome enrichment during library preparation (indicated with text - No enrichment).

The sensitivity of whole-genome enrichment of *C. trachomatis* DNA using SureSelect^XT^ was evaluated using a series of dilutions of *C. trachomatis* genomic DNA extracted from a cultured sample. For each dilution human gDNA was used to bulk the sample to contain 3 μg of starting material prior to library preparation and sequencing. The proportion of sequence reads mapping to a *C. trachomatis* reference genome (F/SW4) was calculated for each dilution (Figure 3). The data showed a saturation of ~90% on-target reads irrespective of the amount of input *C. trachomatis* DNA. From approximately 48,000 *C. trachomatis* input genomes (based on qPCR of the genomic *omcB* gene) we obtained close to 100% coverage of the reference genome with a mean read depth of 20× and around 85% coverage of the reference genome at a mean read depth of 100× (Figure 4). With a ten-fold lower input (4,800 *C. trachomatis* genomes) we obtained 98% coverage of the reference genome but with a lower mean read depth (Figure 4). Using whole-genome enrichment, a high proportion of sequence reads mapping to *C. trachomatis* were obtained from all of the cultured samples and full length genomes were recovered, confirming that our 120-mer RNA oligonucleotide set was capable of enriching for strains from both biovars (Table 1).

**Table 1:** **Overview of raw sequence data obtained from the cultures** ***C. trachomatis*** **samples**

ID	Sample type	Genome copies in 1-2 ml DNA extract	Total input***C. trachomatis***genome copies	Total reads	Mapped reads	Reads mapping to***C. trachomatis***	Serovar/Genotype (***ompA***)
CT_10	Culture	2.3x10^9^	362,346,600	3450960	3058284	88.62%	B
CT_11	Culture	3.5x10^8^	2,098,188,000	3847486	3455294	89.81%	C
CT_12	Culture	8.1x10^9^	683,544,000	5055724	4632017	91.62%	G
CT_13	Culture	1.1x10^9^	1,070,106,000	3923244	3512706	89.54%	H
CT_14	Culture	1.8x10^9^	8,421,480,000	4814146	4524936	93.99%	I
CT_15	Culture	1.4x10^10^	5,587,859,200	4572350	4242802	92.79%	J
CT_16	Culture	1.4x10^10^	1,563,216,000	7734134	7240982	93.62%	L1
CT_17	Culture	2.6x10^9^	1224,048,000	6316494	5681915	89.95%	L2

All 8 samples were sequenced in a MiSeq run. The total input refers to the number of *C. trachomatis* genome copies used as input in the library preparation. The genotyping (*ompA*) was performed in the clinical laboratory.

**Figure 5:**
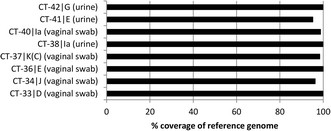
**Coverage of reference genome - consensus sequences obtained directly from clinical samples.** The plot shows the coverage of the reference genome (%) found when aligning the consensus sequences generated from the eight clinical samples that produced good genomic data.

Using whole-genome enrichment, *C. trachomatis* was directly sequenced, without culture, from 10 clinical diagnostic samples containing between 33,000 to >6.8x10^6^*C. trachomatis* genome copies per enrichment reaction. Each sample was sequenced twice on the MiSeq platform and the data were pooled to obtain between 1-50% sequence-reads mapping to *C. trachomatis*. The sequence results are summarised in Table 2. Eight of the ten clinical samples, five vaginal swabs and three urine samples, generated >95% coverage of the reference genome (Figure 5) with a mean read depth between 6x - >400x (Table 2). For all 10 clinical samples, we were able to map the full length *C. trachomatis* plasmid with high mean read depth (Additional file 2). The limit for generating full genomes from vaginal swabs was 551,820 *C. trachomatis* genomes (6,492 genome copies/μl) in 100 μl DNA extract from 200 μl of the original sample, corresponding to a PCR cycle threshold (C_t_) of 27. The limit for generating full genomes from urine samples was 33,320 *C. trachomatis* genomes (392 genome copies/μl) in 100 μl DNA extract from 200 μl of the original sample, corresponding to a C_t_ of 31. Both reference based and *de novo* assembly were performed with the read data from the sequenced clinical samples. The proportion of *C. trachomatis* reads was sufficient for *de novo* assembly of samples CT-33 and CT-38, generating three and ten contigs respectively, whereas the proportion of reads mapping to *C. trachomatis* was suboptimal for *de novo* assembly of six samples (CT-34, CT-36, CT-37, CT-40, CT-41, CT-42), generating between 46-297 contigs. The contigs generated by *de novo* assembly from sample CT-33 were compared to the corresponding consensus sequence extracted after reference based mapping and showed 99.8-100% homology between the sequences (data not shown).

**Table 2:** **Analysis of sequence data from DNA extracted directly from clinical** ***C. trachomatis*** **samples**

ID	Original sample type	C_t_-value (***omcB***)	Genome copies in 100 μl DNA extract	Total input***Chlamydia***genome copies	Total reads	Mapped reads	Reads mapping to***C. trachomatis***	Mean read depth	Coverage of ref. genome	Serovar/Genotype (***ompA***)
CT-33	Vaginal swab	19.3	1.2x10^8^	68,864,400	5,493,094	2,722,903	49.57%	410	99.9%	D
CT-34	Vaginal swab	26.5	1.0x10^6^	868,530	8,016,738	105,030	1.31%	15.8	96.3%	J
CT-35	Vaginal swab	29	2.0x10^5^	168,980	3,740,600	14,129	0.38%	2	59.1%	-
CT-36	Vaginal swab	26.5	1.0x10^6^	864,705	3,731,308	195,331	5.23%	29.4	100.0%	E
CT-37	Vaginal swab	26.2	1.3x10^6^	1,092,760	3,993,426	73,356	1.84%	11	98.4%	C/K
CT-38	Urine	29.4	1.5x10^5^	129,710	4,425,438	194,923	4.40%	29.4	99.9%	Ia
CT-39	Vaginal swab	30.6	7.0x10^4^	59,755	4,454,502	2,975	0.07%	0.4	24.3%	-
CT-40	Vaginal swab	27.2	6.5x10^5^	551,820	7,083,232	110,006	1.55%	16.5	98.8%	Ia
CT-41	Urine	31.5	3.9x10^4^	33,320	4,159,384	41,937	1.01%	6.3	95.2%	E
CT-42	Urine	30.1	1.0x10^5^	86,360	4,590,004	111,826	2.44%	16.9	99.9%	G

All 10 samples were multiplexed and sequenced twice on a MiSeq in two separate runs, after which the data-sets were combined. C_t_-value refers to a cycle threshold obtained by qPCR and were defined in the clinical laboratory. Genome copies in 100 μl DNA extract from 200 μl original sample. The total input refers to the number of *C. trachomatis* genome copies used as input in the library preparation. *In silico* genotyping was performed comparing the *ompA* gene sequence to other known serovars found in GenBank.

**Figure 6:**
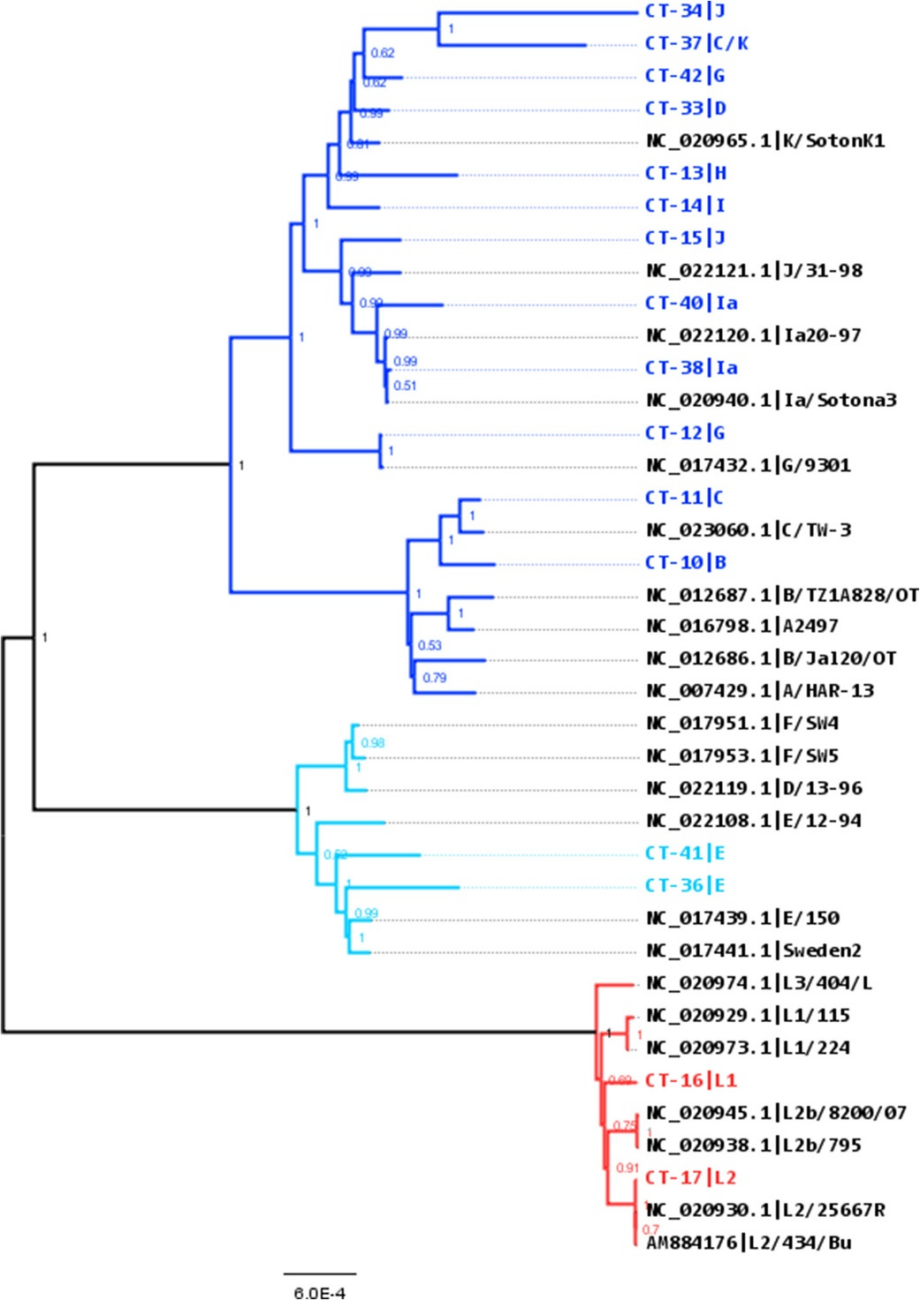
**Neighbour-joining reconstruction of the phylogeny of** ***C. trachomatis*** **.** The figure shows an un-rooted phylogenetic reconstruction of the alignment data including 8 cultured and 8 clinical *C. trachomatis* samples sequenced in this study and 23 *C. trachomatis* genomes obtained from GenBank. The Neighbour-joining tree was constructed with 500 bootstrap replicates using a Jukes Cantor model of evolution and gamma correction for among-site rate variation with four rate categories. The gap/missing data was treated with a site coverage cut-off of 90%. The blue clusters represent the ocular/urogenital biovar (dark blue T2 clade and light blue T1 clade) and the red cluster represents the LGV biovar. The ocular serovars A-C cluster within the dark blue T2 clade. The samples sequenced in this study are highlighted in colours (blue and red) and the GenBank strains are represented in black.

Altogether 16 full consensus genomes (8 clinical samples and 8 cultured samples) generated by reference based mapping were aligned against a collection of 23 *C. trachomatis* genomes retrieved from GenBank. Phylogenetic analysis of the aligned sequences by neighbour joining and maximum likelihood showed identical topology. Two main clusters were identified dividing the *ompA* serovars into the ocular/urogenital and LGV biovars with the ocular/urogenital biovar subdivided into clades T1 and T2 (Figure 6) as previously shown [14]. The *in silico ompA* genotyping of sample CT-37 was inconclusive. From a phylogenetic analysis with the *ompA* gene sequence sample CT-37 was genotyped as serovar C or K (data not shown) but from a phylogenetic analysis using the complete genome, sample CT-37 clustered with the urogenital serovars (D-K) rather than the ocular serovars (A-C) (Figure 6). The samples that sequenced successfully in this study were found throughout the tree, confirming that our method can be used for strains from both biovars. As has previously been described, we found evidence of *C. trachomatis* recombination within the *ompA* gene (Additional file 3) [14].

At least 34 substitutions (single or in combination) have been reported in association with clinical and/or *in vitro C. trachomatis* resistance to antimicrobial drugs (Additional file 4). Although we had no data to suggest that any of the clinical samples showed phenotypic antimicrobial resistance, we examined consensus sequences for the presence of reported mutations. Three mutations in the L22 gene were identified in one sample (CT-36, Additional file 5). L22 mutations have been associated with resistance to macrolide antibiotics when they occur together with two mutations within one of the 23S rRNA genes [33]; however, we found no mutations in the 23S rRNA genes for this sample.

*C. trachomatis* in clinical samples is thought to contain a mix of infectious EBs and replicating RBs. The latter is hypothesised to acquire mutations that confer clinical resistance to antimicrobials [7]. Thus deep sequencing of antimicrobial resistant *C. trachomatis* may reveal a heterogeneous population of resistant and sensitive strains.

While none of our directly sequenced clinical strains showed all mutations necessary for antimicrobial resistance, we were able to evaluate baseline *C. trachomatis* heterogeneity in sample CT-33 which had a mean read depth of >400×. Based on a previous study [20], we called only variants that were present at sites with a minimum read depth of 40× and a minimum average base call quality of 20. A variant count on a minimum of two reads was required with the variant present in both forward and reverse reads and a strand bias within a 20%-80% interval. We identified variant sites to be present in ~0.08% of the genome with a 50:50 ratio of non-synonymous to synonymous mutations, scattered throughout the genome and present for the most part at <5% frequency (Additional file 6). Only six single nucleotide variants causing non-synonymous substitutions were identified at frequencies >5% (Additional file 7). Two genes encoding hypothetical proteins, contained one variant site each, at frequencies of around 10%. Two other genes, encoding a histone H1-like protein HC2 and a candidate inclusion membrane protein, contained two variant sites each at frequencies between 23-28%. The variant sites within the gene encoding the histone H1-like protein HC2 were found to be linked on the same sequence reads, whereas the variant sites in the gene encoding the candidate inclusion membrane protein were not linked. Two further clinical samples, CT-42 at the variant level and CT-34 at the consensus level, were found to contain the same variant in the histone H1-like protein HC2 encoding-gene. Three clinical samples, CT-34 at the variant level and CT-37/CT-40 at the consensus level, contained the variant sites in the inclusion membrane protein encoding-gene. The variable loci in the histone H1-like protein HC2 encoding-gene and reference position 217,000 in the candidate inclusion membrane protein encoding-gene (Additional file 7) were found to be polymorphic in *C. trachomatis* GenBank consensus sequences.

Twenty-one synonymous variant sites within coding regions were identified at frequencies >5% in sample CT-33 (data not shown). Of these, 18 were linked on the same reads mapping within a 200 bp region in the *tufA* gene, which encodes the translation elongation factor Tu. Variant alleles within this region were present in all seven vaginal swab samples and one of the urine samples (CT-42). BLASTn analysis showed high homology between the vaginal *tufA* variant region and *Mobiluncus* and *Mycoplasma hominis*, which can both be found within the normal vaginal microbiota. The *tufA* variant region identified in the urine sample (from male patient) showed high homology to *Lactobacillus casei*, which is a bacterial species present in human gut.

The non-synonymous SNP profiles obtained from the vaginal swabs were compared to the profiles obtained from the urine samples. No non-synonymous SNP patterns were associated with body-compartment (Additional file 8). The vaginal swab sample CT-36 and the urine sample CT-41 were found to contain fewer non-synonymous SNPs compared to the F/SW4 reference, all clustering within the ocular/urogenital T1 clade (Figure 6).

## Discussion

We have shown that hybridising sequence libraries with 120-mer RNA oligonucleotides, designed specifically against the *C. trachomatis* genome, increases the targeted genomic DNA to a level that enable sequencing of full genomes (>95-100% coverage of a reference genome) from clinical specimens at >10-fold higher sensitivity than has previously been reported [18]. The method does not require prior genome amplification and is therefore simpler and has been proven more consistent in success than previous methods [18],[39]. Whole genome sequencing captures all of the genomic information and has the potential to improve our understanding of *C. trachomatis* evolution. The widespread use of antimicrobials and the global increase in antimicrobial resistance among sexually transmitted bacterial infections [40] also argues for robust and reproducible sequence based surveillance using reliable high throughput methodologies.

From our data, the enrichment approach increased the proportion of reads mapping to *C. trachomatis*, enabling full length genomes to be extracted from 80% of the clinical samples without prior genome amplification. This compares favourably with the recovery of only a partial *C. trachomatis* genome from a single vaginal swab sample in a previous study and full genomes from 15-30% clinical samples after genome amplification in another study [39],[18]. The limit of detection has previously been identified to a diagnostic C_t_-value of 23.1, when generating complete genomes directly from clinical samples [18]. Our whole-genome enrichment generated a full consensus genome from a vaginal swab sample with approximately 552,000 genome copies (~6,500 copies/μl and C_t_-value of 27) and from a urine sample with approximately 33,000 genome copies (~400 copies/μl and C_t_-value of 31). Importantly the whole-genome enrichment method did not add mutagenic bias to the sequence data. The bait set, which was designed from known *C. trachomatis* genomes retrieved from GenBank, enriched for *C. trachomatis* from both biovars and from 10 distinct *ompA* serovars. The sequencing read depth was independent of *ompA* serovar, but related to input genome copy number with the two vaginal swab samples that failed to generate full genomes, falling below the current technical threshold of ~552,000 genome copies per enrichment reaction. However, for low titre samples containing <552,000 genome copies, we were able to generate complete plasmid sequences. Finally our data confirm that whole-genome enrichment did not alter the consensus sequence, with high sequence identity between a enriched and un-enriched cultured sample.

We found that recovery of genomes from urine is more efficient than from vaginal swabs, a finding that is explained by the high complexity of vaginal swabs containing large amounts of contaminating DNA from the natural microbiota and the host. This explanation is supported by the finding that reads mapping to the rRNA genes were overrepresented in the vaginal swabs (see coverage plot in Additional file 1) and that these reads also showed homology (identified by BLASTn search) to 16S rRNA in *Lactobacillus iners* and *Lactobacillus cripatus*, both commonly found as part of the vaginal microbiota [41]. Whole-genome enrichment of *C. trachomatis* from vaginal swabs also enriched a highly conserved region from the *tufA* gene homologous to *Mobiluncus* and *Mycoplasma hominis* both found in the vaginal microbiota and which have been associated with bacterial vaginosis (BV) [42],[43]. In contrast, only one of the three urine *C. trachomatis* sequences (all urine samples were from male patients) showed contamination within this conserved region of the *tufA* gene. Here the contaminant showed homology to *Lactobacillus casei* (identified by BLASTn search), which is a known bacteria that can be found in the human gut [44]. *L. casei* can also colonize the vagina when the normal vaginal microbiota is disrupted [45] and will therefore also be a potential contaminant in sequences obtained from vaginal swab samples. This, together with the fact that we recovered <1% sequence reads mapping to *C. trachomatis* from the two low copy number samples that failed to generate complete genomes, underlines the need for further optimisation both technically and computationally to screen out contaminating sequences.

Generation of WGS is useful for understanding the evolution of *C. trachomatis* antimicrobial resistance, an area of increasing importance for the management of sexually transmitted bacteria. None of the subjects from whom we obtained samples had evidence of resistant bacteria. Nonetheless, the identification of three fixed SNPs in the L22 gene from sample CT-36, which have been associated with macrolide resistance when in combination with two mutations in the 23S rRNA gene [33], highlights the potential use of WGS of *C. trachomatis* for stratification of treatment options. In this case avoiding treatment with macrolides would potentially reduce the chance of acquiring 23S rRNA mutations and, consequently, macrolide resistance. Putative *C. trachomatis* antimicrobial resistance is postulated to arise from heterogeneous populations [7]. Alternatively, compartmentalisation of bacteria with some being exposed to antimicrobials and becoming resistant while others are sequestered and remain unexposed and sensitive, would produce a similar outcome. Because of the high read depth generated, next generation sequencing methods provide an opportunity to interrogate variant data, including the presence of low-level resistance mutations. We have previously shown, using a highly heterogeneous herpesvirus vaccine, that for allele frequencies >1%, whole-genome enrichment methods do not change the population structure [20]. For sample CT-33 where the mean read depth was >400×, we found 870 variant sites of which more than 86% were present at frequencies ≤5%. Of the 27 synonymous and 6 non-synonymous single nucleotide variants that occurred at frequencies >5%, 18 synonymous mutations were found to be due to contaminating *tufA* gene sequences from other microorganisms present in the natural microbiota and 15 being due to *C. trachomatis* mutations. Four non-synonymous variant sites were found to be natural polymorphisms, as the variants were fixed in some genomes but not in others. Our findings raise the possibility that these positions are heterogeneous *in vivo.* However, since most available sequences for this region are from cultured isolates, which are much less heterogeneous, sequencing of more clinical specimens is needed. None of the 15 confirmed variant sites were located in genes that have been associated with antimicrobial resistance. However, the low background variation could offer the opportunity for easy identification of heterogeneous resistance mutations from variant read data and this will be explored further with suitable sample sets.

## Conclusions

We have shown that novel whole-genome enrichment increase the sensitivity by >10-fold when performing WGS of *C. trachomatis* directly from clinical specimens. The method is fully automatable and provides the potential for high-throughput sequencing of this intracellular bacterium and the generation of large datasets for better understanding of diversity, evolution and antimicrobial resistance. The method might be less applicable to bacteria exploiting lateral gene transfer as means of evolution, but has great potential to be applied on other obligate intracellular or fastidious pathogens with a clonal population structure.

## Authors' contributions

MTC carried out the sequence data analysis and drafted the manuscript. ACB carried out the whole-enrichment and sequencing. SK assisted in the sequence data analysis, particular the recombination analysis. HJT extracted DNA from the samples and performed quantitative PCR. RW coordinated the study. JRB assisted in sample collection, coordination and quantitative PCR. JH assisted in RNA baits design. MJH, SS, JD, CYWT supplied the samples. KE-J assisted in the sequence data analysis. DPD assisted in RNA baits design and sequence data analysis, particular the multiple sequence alignments. JB conceived the study and helped to draft the manuscript. All authors read and approved the manuscript.

## Additional files

## Electronic supplementary material

Additional file 1: Default parameters using CLC Genomic Workbench - sample CT-33|D (extracted from a vaginal swab sample) used as example.(PDF 166 KB)

Additional file 2: Recovery of complete plasmid sequence directly from clinical specimens. All 10 samples were multiplexed and sequenced twice on a MiSeq in two separate runs, after which the data-sets were combined. (PDF 98 KB)

Additional file 3: **Recombination in the** ***ompA*** **gene.** The plot illustrates the similarity of the *ompA* gene sequence from sample CT-38 to the *ompA* gene sequences from each of the clinical samples. Breakpoints were identified at positions 301 and 967 and are highlighted by the asterisks. (PDF 172 KB)

Additional file 4: **Summary of known antimicrobial resistance mutations in** ***C. trachomatis.*** Summary of mutations associated with antibiotic resistance in *Chlamydia trachomatis.*(PDF 38 KB)

Additional file 5: Mutations associated with antimicrobial resistance identified in one clinical sample (CT-36|E). Mutations associated with antibiotic resistance identified in sample CT-36|E. (PDF 111 KB)

Additional file 6: **Non-synonymous and Synonymous Variants (>0% - <50%).** Non-synonymous and synonymous variants in sample CT-33|D at frequencies between >0% - <50% plotted against position in the *C. trachomatis* genome. (PDF 156 KB)

Additional file 7: Variants >5% frequency found within one clinical sample (CT-33|D). Non-synonymous Single Nucleotide Variants >5% frequency. (PDF 165 KB)

Additional file 8: **Genome Atlas - comparing SNPs differences identified in vaginal swabs and urine samples.** The genome atlas illustrates the non-synonymous SNP differences found between eight clinical C*hlamydia trachomatis* samples processed with whole-genome enrichment and the GenBank reference strain F/SW4 (Accession no. NC_017951.1). The two outer tracks shown in green illustrate the open reading frames (ORFs) annotated in the reference strain with forward and reverse orientation respectively. The red tracks show all the non-synonymous SNP differences found between the vaginal swab samples and the reference strain. The blue tracks show all the non-synonymous SNP differences found between the urine samples and the reference strain. (PDF 3 MB)

Below are the links to the authors’ original submitted files for images.

Authors’ original file for figure 1

Authors’ original file for figure 2

Authors’ original file for figure 3

Authors’ original file for figure 4

Authors’ original file for figure 5

Authors’ original file for figure 6

Authors’ original file for figure 7

Authors’ original file for figure 8
