# Supplementary material for: Whole-genome enrichment and sequencing of Chlamydia trachomatisdirectly from clinical samples
Source: BMC Infect Dis. 2014 Nov 12;14:591. doi: 10.1186/s12879-014-0591-3 (PMC4233057; doi:10.1186/s12879-014-0591-3)

Additional file 1: Default parameters using CLC Genomic Workbench – sample CT-33lD (extracted from a vaginal swab sample) used as example

Examples from quality report:

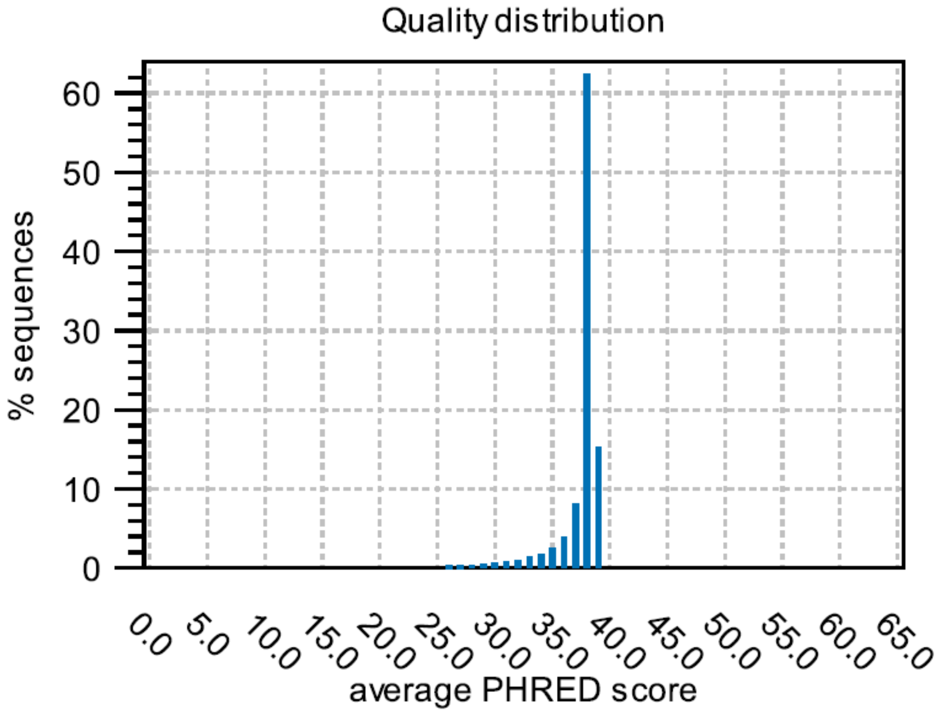

Distribution of average sequence qualitie scores. The quality of a sequence is calculated as the arithmetic mean of its base qualities.
x: PHRED-score
y: number of sequences observed at that qual. score normalized to the total number of sequ ences

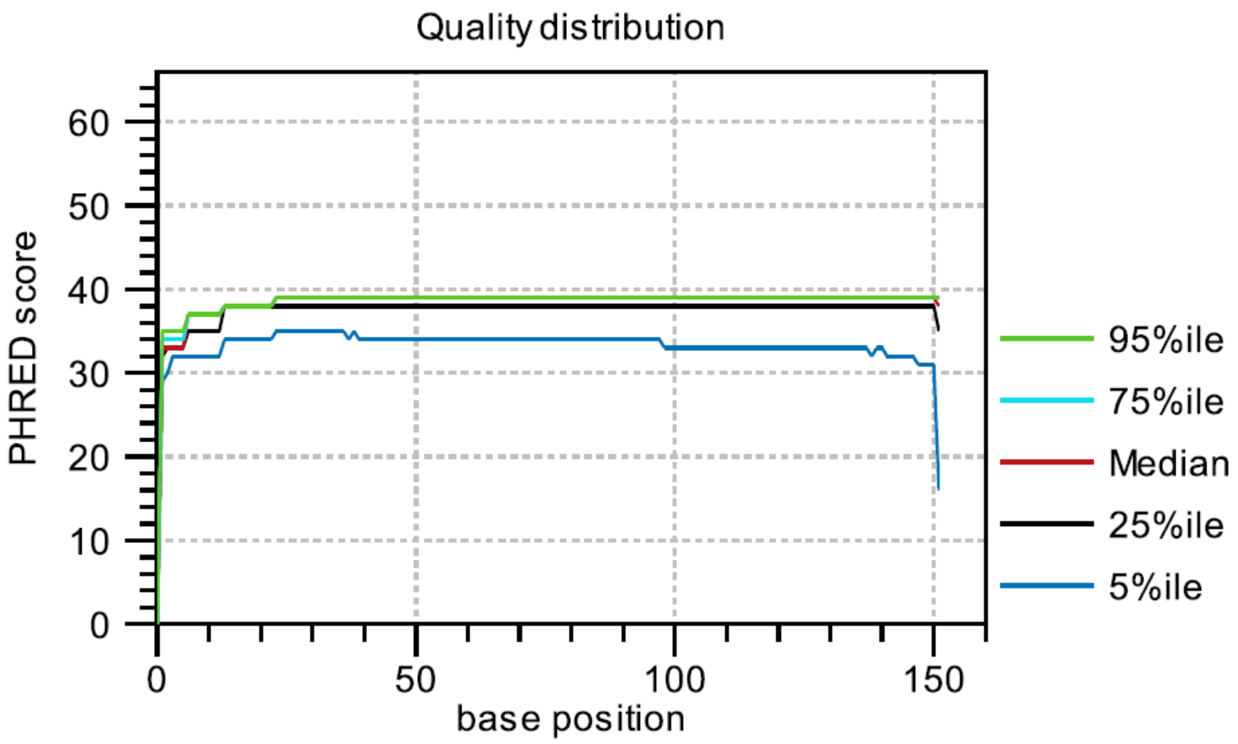


```
Base-quality distribution along the base positions.
x: base position
y: median & percentiles of quality scores observed at that base position
```

Parameters for trim of sequnces:

- Trim ambiguous reads
- Quality trim with quality trim limit of 0.05 = Phred 13

For sample CT-33: A total of 5,493,572 sequence reads were processed of which 239 sequences have been completely removed during trimming and 585,041 nucleotides have been trimmed altogether.

Parameters for mapping:

- References NC_017951 F_SW4
- Masking mode: No masking (for determination of mean read depth the rRNA regions were mapped for sequences obtained from vaginal swab samples).
- Mismatch cost 2
- Insertion cost 3
- Deletion cost 3
- Length fraction 0.5
- Similarity fraction 0.8
- Global alignment No
- Auto-detect paired distances Yes
- Non-specific match handling Map randomly

For samples CT-33: Reads mapped: 2,815,829 of 5,493,094 Estimated paired distance range(s): 63 to 468 bp

Parameters for extracting consensus sequence:

- Threshold 4
- Action Insert 'N' ambiguity symbols
- Conflict resolution strategy Vote

Coverage plot of sample CT-33:

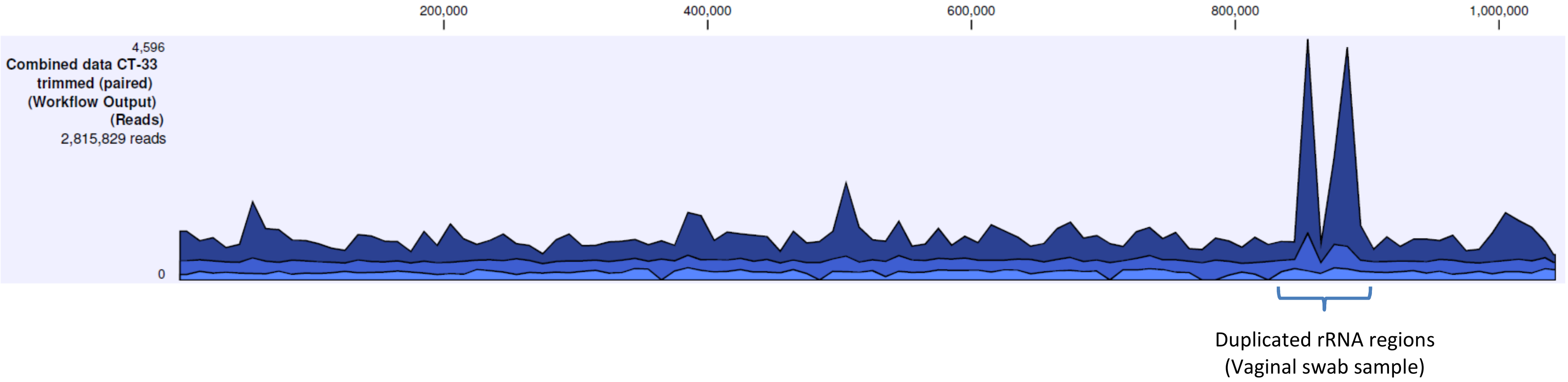

Supplement: Supplementary file 1 — Additional file 1: Default parameters using CLC Genomic Workbench - sample CT-33|D (extracted from a vaginal swab sample) used as example.(PDF 166 KB) [file 12879_2014_591_MOESM1_ESM.pdf]
