# Supplementary material for: Whole-genome enrichment and sequencing of Chlamydia trachomatisdirectly from clinical samples
Source: BMC Infect Dis. 2014 Nov 12;14:591. doi: 10.1186/s12879-014-0591-3 (PMC4233057; doi:10.1186/s12879-014-0591-3)
Supplement: Supplementary file 2 — Additional file 2: Recovery of complete plasmid sequence directly from clinical specimens. All 10 samples were multiplexed and sequenced twice on a MiSeq in two separate runs, after which the data-sets were combined. (PDF 98 KB) [file 12879_2014_591_MOESM2_ESM.pdf]

Additional file 2: Recovery of complete plasmid sequence directly from clinical specimens

ID	Plasmid copies/µl	Reads mapping to *C. trachomatis* plasmid	Mean read depth	Coverage of reference *C. trachomatis* plasmid
CT-33	4,350,790	37.91%	41945	100%
CT-34	45,417	2.31%	3727	100%
CT-35	16,948	0.20%	147	100%
CT-36	80,826	1.06%	797	100%
CT-37	54,154	0.80%	642	100%
CT-38	4,698	0.34%	301	100%
CT-39	4,056	0.12%	104	100%
CT-40	22,388	4.08%	5822	100%
CT-41	2,367	0.11%	91	100%
CT-42	5,093	0.30%	277	100%
