# Supplementary figures and images for: Whole-genome enrichment and sequencing of Chlamydia trachomatisdirectly from clinical samples
Source: BMC Infect Dis. 2014 Nov 12;14:591. doi: 10.1186/s12879-014-0591-3 (PMC4233057; doi:10.1186/s12879-014-0591-3)

### Additional file 3: Recombination in the *ompA* gene

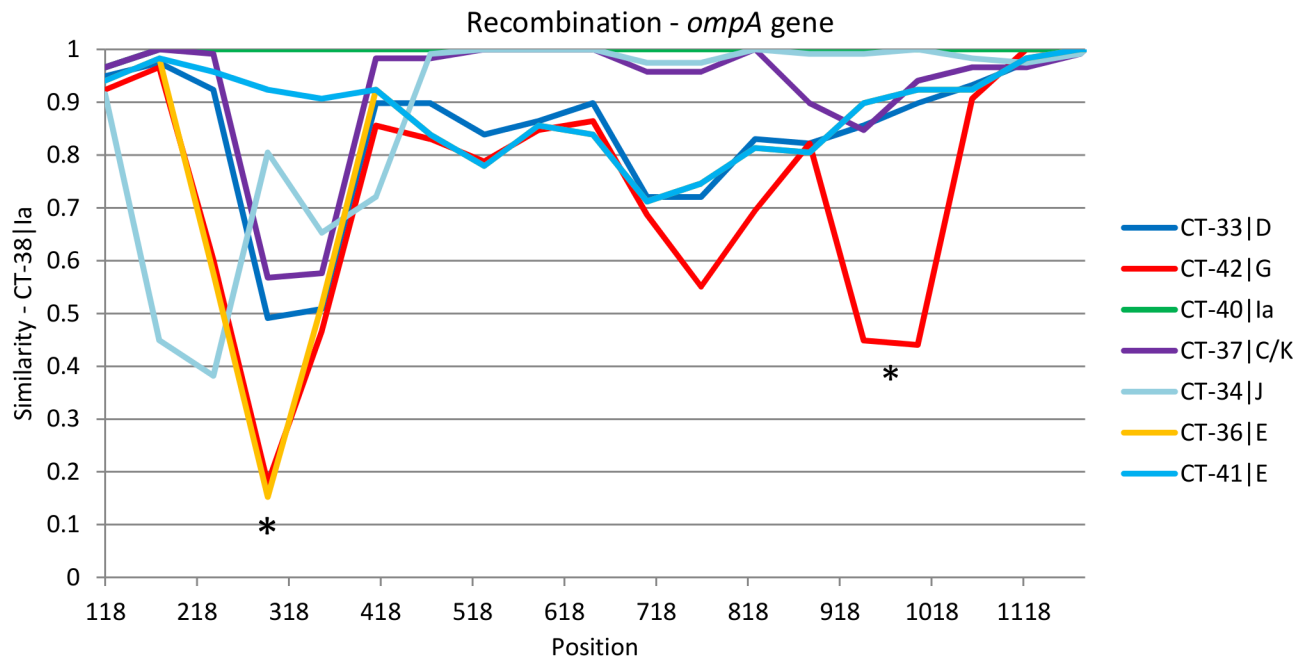

Supplement: Supplementary file 3 — Additional file 3: Recombination in the ompA gene. The plot illustrates the similarity of the ompA gene sequence from sample CT-38 to the ompA gene sequences from each of the clinical samples. Breakpoints were identified at positions 301 and 967 and are highlighted by the asterisks. (PDF 172 KB) [file 12879_2014_591_MOESM3_ESM.pdf]

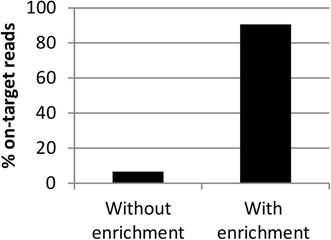

Supplement: Supplementary file 9 — Authors’ original file for figure 1 [file 12879_2014_591_MOESM9_ESM.gif]

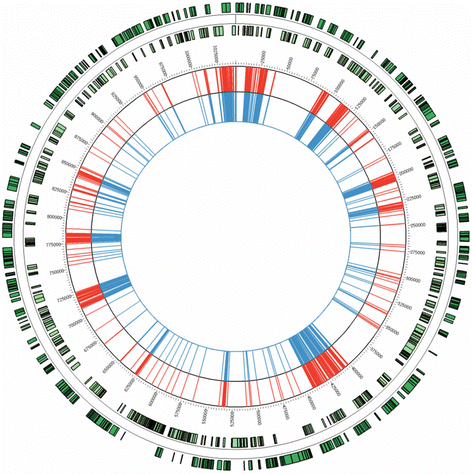

Supplement: Supplementary file 10 — Authors’ original file for figure 2 [file 12879_2014_591_MOESM10_ESM.gif]

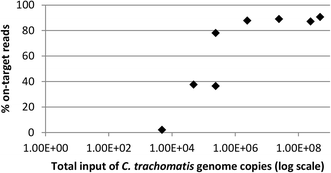

Supplement: Supplementary file 11 — Authors’ original file for figure 3 [file 12879_2014_591_MOESM11_ESM.gif]

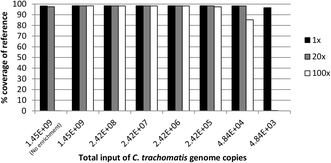

Supplement: Supplementary file 12 — Authors’ original file for figure 4 [file 12879_2014_591_MOESM12_ESM.gif]

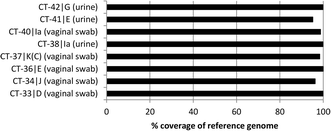

Supplement: Supplementary file 13 — Authors’ original file for figure 5 [file 12879_2014_591_MOESM13_ESM.gif]

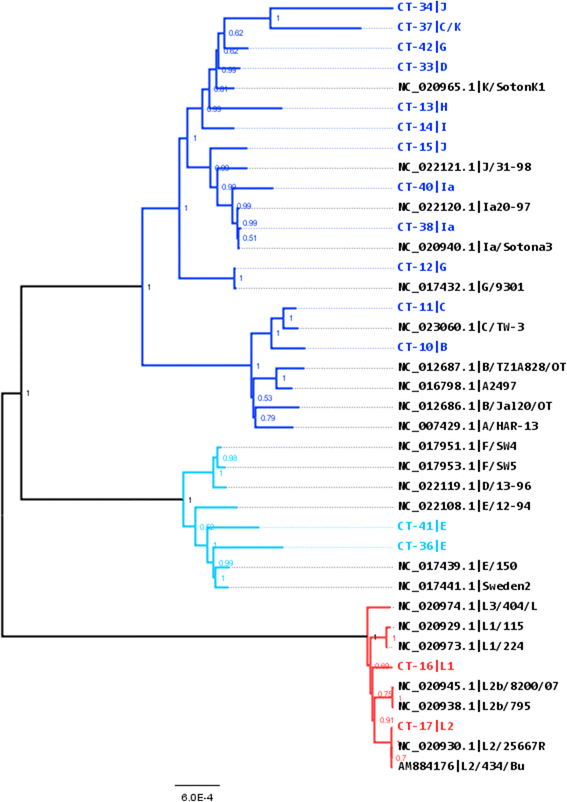

Supplement: Supplementary file 14 — Authors’ original file for figure 6 [file 12879_2014_591_MOESM14_ESM.gif]

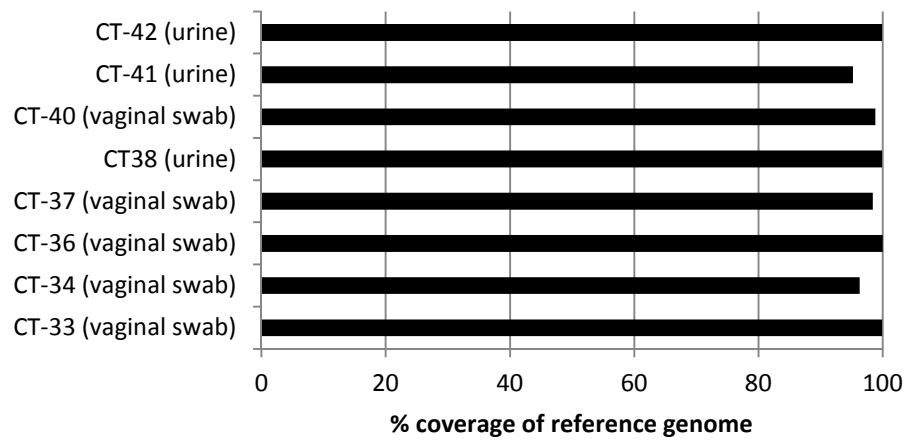

Supplement: Supplementary file 15 — Authors’ original file for figure 7 [file 12879_2014_591_MOESM15_ESM.pdf]

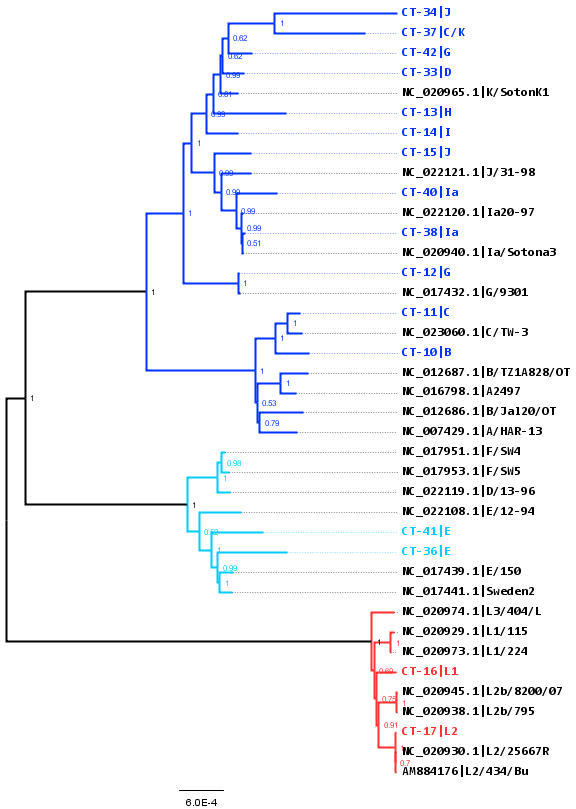

Supplement: Supplementary file 16 — Authors’ original file for figure 8 [file 12879_2014_591_MOESM16_ESM.png]
