# Supplementary material for: Whole-genome enrichment and sequencing of Chlamydia trachomatisdirectly from clinical samples
Source: BMC Infect Dis. 2014 Nov 12;14:591. doi: 10.1186/s12879-014-0591-3 (PMC4233057; doi:10.1186/s12879-014-0591-3)
Supplement: Supplementary file 4 — Additional file 4: Summary of known antimicrobial resistance mutations in C. trachomatis. Summary of mutations associated with antibiotic resistance in Chlamydia trachomatis.(PDF 38 KB) [file 12879_2014_591_MOESM4_ESM.pdf]

| Drug | Gene | Mutation 1 | Mutation 2 | Mutation 3 | Substitution | Coding residue/gene nucleotide position |
|---|---|---|---|---|---|---|
| SAH | hemG | G>A | | | glycine - serine | 58 |
| Tetracycline | ompB/porB | C>T | | | stop codon | 81 |
| MDQA | secY | G>T | | | alanine - serine | 45 |
| MDQA | secY | G>C | | | alanine - proline | 246 |
| MDQA | secY | G>C | | | alanine - proline | 420 |
| Macrolide | 23S rRNA | A>G | A>G | T>C | | 2037, 2039, 2591 |
| Macrolide | 23S rRNA | T>C | | | | 2591 |
| Macrolide | 23S rRNA | A>C | | | | 2038 |
| Macrolide | 23S rRNA | A>C | T>C | | | 2038, 2591 |
| Macrolide | 23S rRNA | A>C | T>C | | | 2038, 2591 |
| | L22 | G>A | C>T | T>C | glycine - serine, arginine - cysteine, valine - alanine | 154, 193, 230 ($52^{aa}$, $65^{aa}$, $77^{aa}$) |
| Macrolide | rplD (L4) | C>A | | | glutamine - lysine | 66 |
| Fluoroquinolones | gyrA (QRDR) | G>T | | | serine - isoleucine | 83 |
| Moxifloxacin | gyrA (QRDR) | A/T>T/C | | | serine - isoleucine | 83 |
| Moxifloxacin | gyrA (QRDR) | T/C>G/C | | | serine - argenine | 83 |
| Rifampicin | rpoB | C>A | | | glutamine - lysine | 458 |
| Rifampicin | rpoB | C>T | | | histidine - tyrosine | 471 |
| Rifampicin | rpoB | C>G | | | histidine - asparagine | 471 |
| Rifampicin | rpoB | A/T/C>G | | | isoleucine - methionine | 517 |
| Rifampicin | rpoB | A/T/C>G | T>C | | isoleucine - methionine, valine - alanine | 517, 466 |
| Rifampicin | rpoB | A/T/C>G | G>A | | isoleucine - methionine, aspartic acid - asparagine | 461, 517 |
| Rifampicin | rpoB | A/T/C>G | A>G | | isoleucine - methionine, histidine - asparagine | 471, 517 |
| Rifampicin | rpoB1 | A>G | | | aspartic acid - glycine (leucine) | 516 |
| Rifampicin | rpoB | C>T | | | alanine-valine | 467 |
| GM6001 and TAPI | cPDF | C>A | | | 5' end of non-coding region | |

SAH: Salicylidene acyldrazides
MDQA: (3-methoxyphenyl)-(4,4,7-trimethyl-4,5-dihydro-1H-[1,2]dithiolo[3,4-C]quinolin-1-ylidene)amine
Macrolide: erythromycin, azithromycin, josamycin
GM6001, TAPI: Hydroxamic-acid-based matrix metalloprotease inhibitors
