# Supplementary material for: Whole-genome enrichment and sequencing of Chlamydia trachomatisdirectly from clinical samples
Source: BMC Infect Dis. 2014 Nov 12;14:591. doi: 10.1186/s12879-014-0591-3 (PMC4233057; doi:10.1186/s12879-014-0591-3)
Supplement: Supplementary file 5 — Additional file 5: Mutations associated with antimicrobial resistance identified in one clinical sample (CT-36|E). Mutations associated with antibiotic resistance identified in sample CT-36|E. (PDF 111 KB) [file 12879_2014_591_MOESM5_ESM.pdf]

Additional file 5: Mutations associated with antimicrobial resistance identified in one clinical sample (CT-36|E)

Gene	Nucleotide Position	Ref	Allele	Count	Coverage	Frequency (%)	Strand balance	Average quality	Coding region change	Amino acid change
L22	154	G	A	11	11	100	0.4	38	154G>A	Gly52Ser
L22	193	C	T	17	18	94.44	0.5	38.35	193C>T	Arg65Cys
L22	230	T	C	13	14	92.86	0.5	38.14	230T>C	Val77Ala
