# Supplementary material for: Whole-genome enrichment and sequencing of Chlamydia trachomatisdirectly from clinical samples
Source: BMC Infect Dis. 2014 Nov 12;14:591. doi: 10.1186/s12879-014-0591-3 (PMC4233057; doi:10.1186/s12879-014-0591-3)

Additional file 6: Non-synonymous and Synonymous Variants (>0% - <50%)

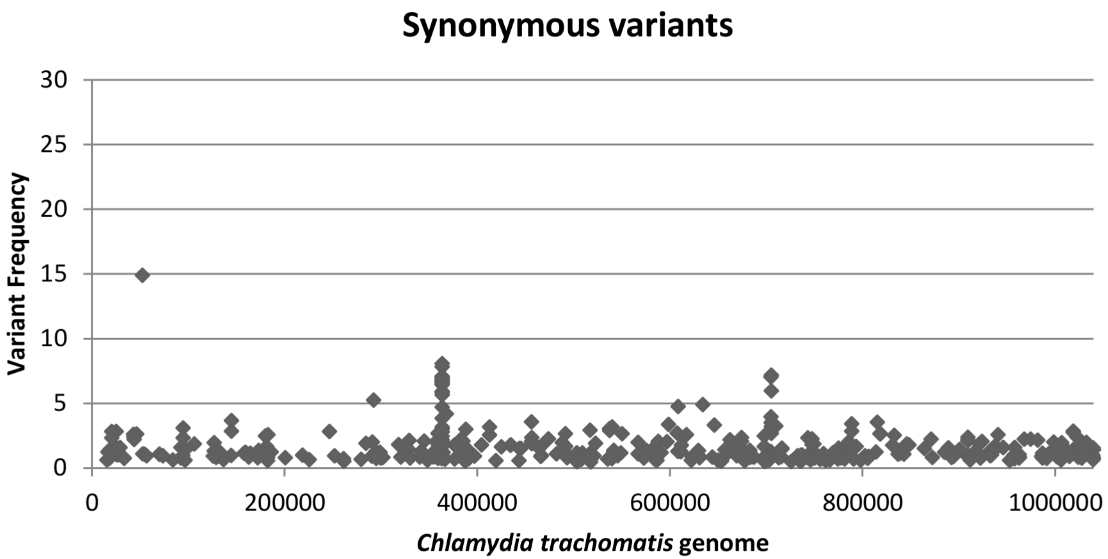

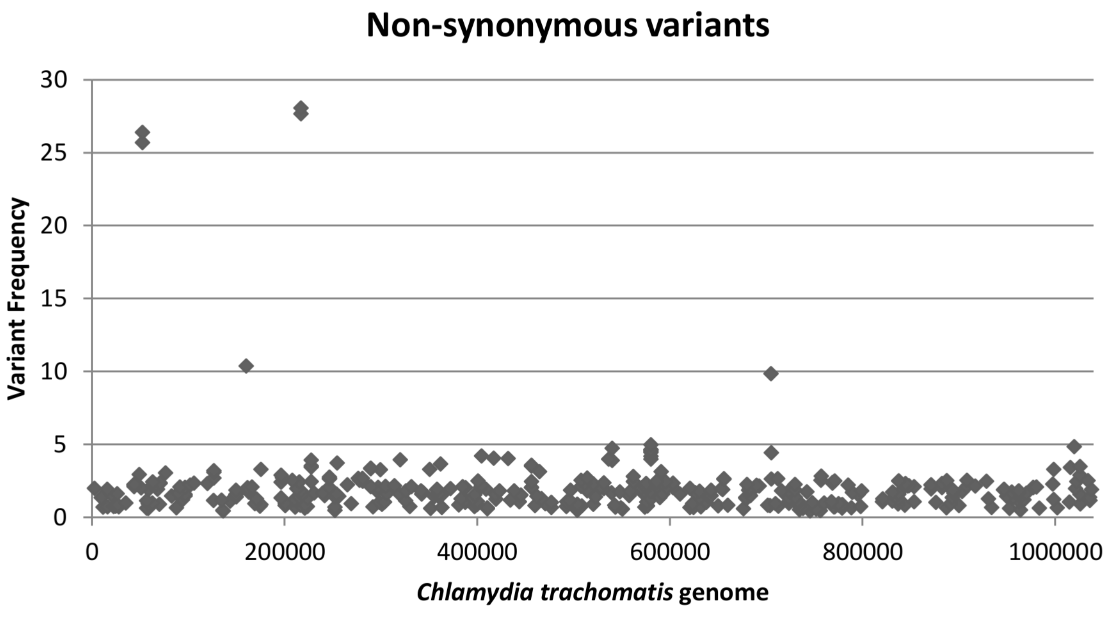

Supplement: Supplementary file 6 — Additional file 6: Non-synonymous and Synonymous Variants (>0% - <50%). Non-synonymous and synonymous variants in sample CT-33|D at frequencies between >0% - <50% plotted against position in the C. trachomatis genome. (PDF 156 KB) [file 12879_2014_591_MOESM6_ESM.pdf]
