# Supplementary material for: Whole-genome enrichment and sequencing of Chlamydia trachomatisdirectly from clinical samples
Source: BMC Infect Dis. 2014 Nov 12;14:591. doi: 10.1186/s12879-014-0591-3 (PMC4233057; doi:10.1186/s12879-014-0591-3)
Supplement: Supplementary file 7 — Additional file 7: Variants >5% frequency found within one clinical sample (CT-33|D). Non-synonymous Single Nucleotide Variants >5% frequency. (PDF 165 KB) [file 12879_2014_591_MOESM7_ESM.pdf]

**Additional file 7: Variants >5% frequency found within one clinical sample (CT-33|D)**

| Ref. Position | Allele in CT-33\|D consensus | Allele variant | Count | Coverage | Frequency | Forward/ Reverse balance | Average quality | Annotation | Amino acid change | Description |
|---|---|---|---|---|---|---|---|---|---|---|
| 704879 | C | A | 22 | 223 | 9.87 | 0.32 | 37.19 | FSW4_6311 | Leu779Met | Hypothetical protein |
| 160169 | C | T | 37 | 356 | 10.39 | 0.49 | 37.99 | FSW4_1431 | Thr168Ile | Hypothetical protein |
| 52335 | C | T | 83 | 323 | 25.7 | 0.42 | 37.42 | FSW4_0471 | Ala77Val | Histone H1-like protein HC2 |
| 52332 | T | C | 84 | 318 | 26.42 | 0.42 | 37.57 | FSW4_0471 | Val76Ala | |
| 217004 | T | C | 49 | 177 | 27.68 | 0.39 | 37.55 | FSW4_1931 | Ile44Val | Candidate inclusion membrane protein |
| 217000 | A | C | 48 | 171 | 28.07 | 0.38 | 38.22 | FSW4_1931 | Val45Gly | |
