# Supplementary material for: Whole-genome enrichment and sequencing of Chlamydia trachomatisdirectly from clinical samples
Source: BMC Infect Dis. 2014 Nov 12;14:591. doi: 10.1186/s12879-014-0591-3 (PMC4233057; doi:10.1186/s12879-014-0591-3)

Additional file 8: Genome Atlas – comparing SNPs differences identified in vaginal swabs and urine samples

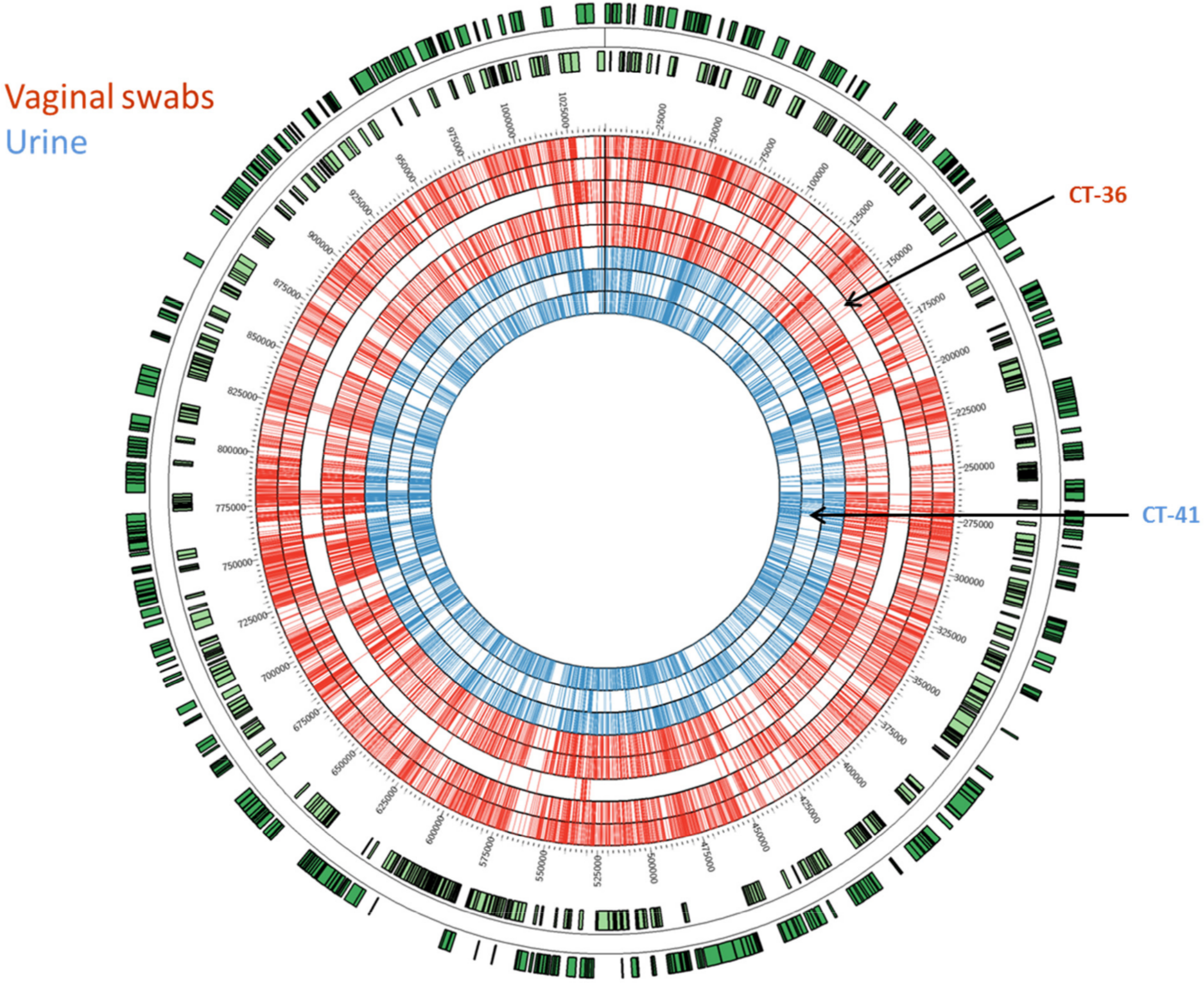

Supplement: Supplementary file 8 — Additional file 8: Genome Atlas - comparing SNPs differences identified in vaginal swabs and urine samples. The genome atlas illustrates the non-synonymous SNP differences found between eight clinical Chlamydia trachomatis samples processed with whole-genome enrichment and the GenBank reference strain F/SW4 (Accession no. NC_017951.1). The two outer tracks shown in green illustrate the open reading frames (ORFs) annotated in the reference strain with forward and reverse orientation respectively. The red tracks show all the non-synonymous SNP differences found between the vaginal swab samples and the reference strain. The blue tracks show all the non-synonymous SNP differences found between the urine samples and the reference strain. (PDF 3 MB) [file 12879_2014_591_MOESM8_ESM.pdf]
